# Supplementary material for: From Gene to Transcript and Peptide: A Deep Overview on Non-Specific Lipid Transfer Proteins (nsLTPs)
Source: Antibiotics (Basel). 2023 May 21;12(5):939. doi: 10.3390/antibiotics12050939 (PMC10215178; doi:10.3390/antibiotics12050939)
Supplement: Supplementary file 1 [file antibiotics-12-00939-s001.zip › Supplementary Figure 1.pdf]

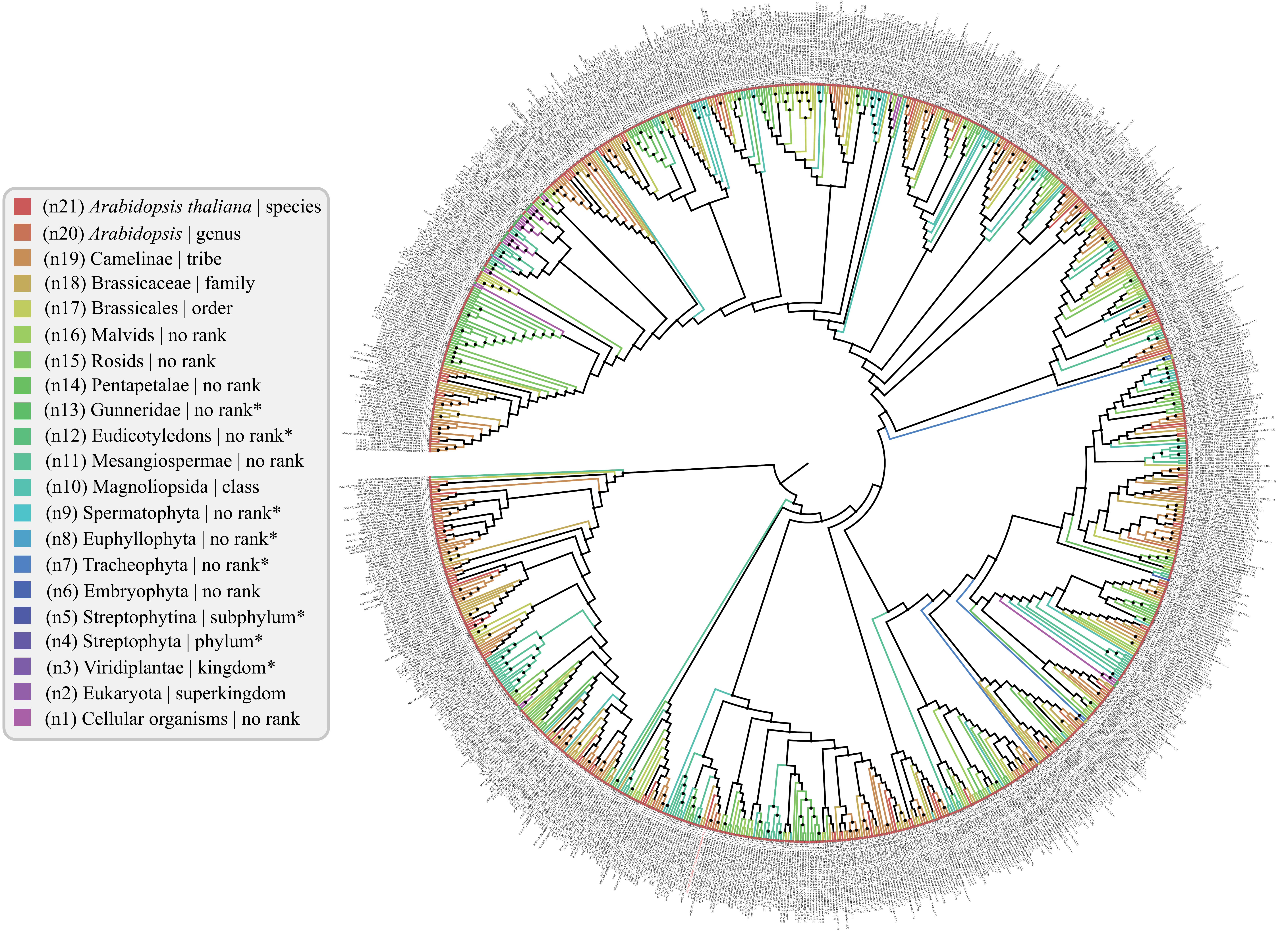
(n21) *Arabidopsis thaliana* | species
(n20) *Arabidopsis* | genus
(n19) Camelinae | tribe
(n18) Brassicaceae | family
(n17) Brassicales | order
(n16) Malvids | no rank
(n15) Rosids | no rank
(n14) Pentapetalae | no rank
(n13) Gunneridae | no rank*
(n12) Eudicotyledons | no rank*
(n11) Mesangiospermae | no rank
(n10) Magnoliopsida | class
(n9) Spermatophyta | no rank*
(n8) Euphyllophyta | no rank*
(n7) Tracheophyta | no rank*
(n6) Embryophyta | no rank
(n5) Streptophytina | subphylum*
(n4) Streptophyta | phylum*
(n3) Viridiplantae | kingdom*
(n2) Eukaryota | superkingdom
(n1) Cellular organisms | no rank
