# Supplementary material for: From Gene to Transcript and Peptide: A Deep Overview on Non-Specific Lipid Transfer Proteins (nsLTPs)
Source: Antibiotics (Basel). 2023 May 21;12(5):939. doi: 10.3390/antibiotics12050939 (PMC10215178; doi:10.3390/antibiotics12050939)
Supplement: Supplementary file 1 [file antibiotics-12-00939-s001.zip › Supplementary Material 1_ok.pdf]

Supplementary Material 1. The methodology used for nsLTP manuscripts' mining and for carrying out the original analyses presented in this work.

1. 'Omics studies for plant nsLTPs' section

1.1 Data mining for published nsLTP manuscripts

Works that address the nsLTPs theme in different species were searched in the PubMed database (https://pubmed.ncbi.nlm.nih.gov/), comprising more than 34 million citations for biomedical literature from MEDLINE, life science journals, and online books.

For data mining, the following keywords were used: 'nsLTPs AND plants', 'LTPs AND plants', 'nsLTPs AND genome-wide AND plants', 'LTPs AND genome-wide AND plants', 'nsLTPs AND omics AND plants', 'LTPs AND omics AND plants'.

1.2 nsLTPs genome mining and characterization

Reference genomes and conceptual proteomes of 12 plant species available in Phytozome database (https://phytozome.jgi.doe.gov) were selected for nsLTPs search. Four genomes regarded lower plants (**Table 2**): one Bryophyte [*Marchantia polymorpha*, family Marchantiaceae] and two Pteridophyte [*Ceratopteris richardii*, Pteridaceae; *Selaginella moellendorffii* (v.1), Selaginellaceae] and one Gymnosperm (*Thuja plicata*, Cupressaceae). The remaining plants included angiosperm species of different families (**Table 2**): *Gossypium hirsutum* (Malvaceae); *Lactuca sativa* (Asteraceae); *Manihot esculenta* (Euphorbiaceae); *Mimulus guttatus* (Phrymaceae); *Populus trichocarpa* (Salicaceae); *Sinapis alba* (Brassicaceae); *Solanum tuberosum* (Solanaceae); *Spinacea oleracea* (Amaranthaceae).

Three strategies were used for nsLTPs mining:

[1] Data mining using RegEx (Regular Expression) patterns. Homemade sequence spacing patterns - specifically, eight conserved cysteine residues (8CM) domain - for nsLTP genes grouped by classes were used;

[2] HMM (Hidden Markov Model), using the HMMER package (Eddy 2009). For HMM search (cut-off < $1e^{-5}$), the default parameters were adopted. The HMM profiles PF14368 and PF00234 (from PFAM database) were used as queries;

[3] Local sequence alignment using BLASTp (Basic Alignment Search Tool; cut-off < $1e^{-5}$), using nsLPTs sequences (from a diverse species pool) available at Uniprot, NCBI and PhytAMP databases as templates.

All recovered sequences were characterized for nsLTP domain (8CM), using the CD-Search tool (https://www.ncbi.nlm.nih.gov/Structure/bwrpsb/bwrpsb.cgi).

1.3 Neighbor Joining (NJ) analysis

The NJ tree was constructed with nsLTP domain (8CM) sequences inferred by the CD-Search tool (https://www.ncbi.nlm.nih.gov/Structure/bwrpsb/bwrpsb.cgi). The twelve plant species (see 1.2 item above) were used. Additionally, previously classified and characterized nsLTPs (Eldstam et al. 2011) seed-sequences from *Arabidopsis thaliana* (Fonseca-García et al. 2021) and *Medicago truncatula* (Fonseca-García et al. 2021) were employed to assist the classification of the sequences under study. Hierarchical clusters were inferred by NJ method, using the ClustalX2 program (Larkin et al. 2007) resampled to 1000 bootstrap replicates. The phenetic tree was visualized with the iTOL program (https://itol.embl.de/).

1.4 Analysis of nsLTPs genomic expansion mechanisms

We choose the 12 plant genomes listed in nsLTPs mining and characterization in available genomes to analyze nsLTPs expansion mechanisms. Additionally, we added soybean 120 nsLTPs loci analyzed by Fonseca-García et al. (2021).

The Multiple Collinearity Scan toolkit (MCScanX; Wang et al. 2012b) package with downstream analysis mode 'duplicate_gene_classifier' was applied to classify the origins of the duplicated nsLTP loci. The following procedure was used by the MCScanX tool to assign the duplication mechanisms: (1) All genes were initially classified as 'singletons' (i.e., presupposes no duplicates within the genome) and assigned gene ranks according to their order of appearance along chromosomes; (2) BLASTp results were evaluated, and the genes with BLASTp hits to other genes were re-labeled as 'dispersed duplicates'; (3) In any BLASTp hit, two genes were re-labeled as 'proximal duplicates' if they had a difference of gene rank < 20; (4) In any BLASTp hit, two genes were re-labeled as 'tandem duplicates' if they had a difference of gene rank = 1; (5) MCScanX was executed. Genes anchored in collinear blocks were re-labeled as 'WGD/segmental'; (6) So, if a gene appeared in multiple BLASTp hits, it was assigned a unique class according to the order of priority: whole-genome/segmental > tandem > proximal > dispersed.

1.5 Pearson correlation coefficient calculation

The data were analyzed for normal distribution using the Shapiro-Wilk Test of normality (W) and homogeneity (Levene's Test). The correlation between genome size and nsLTPs number was calculated by the COR function, native to the ‘R’ programing language.

2. ‘Evolution’ section

2.1 Lowest Common Ancestor (LCA) analysis

To taxonomically characterize nsLTPs using LCA method, amino acid sequences of all nine nsLTP groups (Edstam et al. 2011) were used for a BLASTp search against 224,211,842 protein sequences from 117,030 organisms in RefSeq database (O’Leary et al. 2016). The RefSeq-NCBI accession number of all nsLTP proteins obtained after BLASTp search were used in TaxOnTree tool (http://bioinfo.icb.ufmg.br/taxontree/#x) for automatic incorporation of taxonomic information of each sequence in a phylogenetic three, allowing recognition of LCA candidates. From RefSeq-NCBI accession number, the TaxOnTree (I) retrieves sequences; (II) aligns them using Muscle (Edgar 2004); (III) infers the phylogenetic tree using the FastTree method (Price et al. 2009); (IV) conducts the taxonomic assignment; (V) determines the LCA; and (VI) colors the tree, exporting the result in nexus format. These steps allowed the generation of a phylogenetic tree including all taxa with nsLTPs identified on RefSeq, from diverse organisms and complexity levels, including *Arabidopsis thaliana* lineage.

3. 'Structural proteomics’ section

3.1 nsLTPs modelling and principal component analysis (PCA)

Initially, sequences provided by Boutrot et al. (2008) (**Supplementary Material 3**) and Edstam et al. (2011) (**Supplementary Material 4**) were selected and their theoretical models were solved using Alpha-Fold2, a computational method that can regularly predict protein structures with atomic precision, even in the cases where no similar structure is known (Jumper et al. 2021). The quality of the theoretical models was evaluated by observing the depth and diversity of the MSA, in addition to a per-residue confidence score (pLDDT) and predicted aligned error (PAE).

Then, two PCA approaches were performed. The first, using the xyz coordinates of the α-carbons of the theoretical models, called structural PCA; and the second, using as a basis the physicochemical individual characteristics of each amino acid residue (net charge, disorder propensity, hydrophobicity, molecular weight, disulfide potential and occupancy), denominated physicochemical properties' PCA, according to the instructions of Shafee and Anderson (2019), using [R] prcomp.

4. 'nsLTPs transcriptional expression: soybean as a case study' section

4.1 *nsLTPs expression in soybean: identification and analysis*

Loci encoding GmnsLTPs (*Glycine max* nsLTPs) were recovered from the work by Fonseca-García et al. (2021). These authors provided the loci ID [Phytozome database (v12.1.6; https://phytozome.jgi.doe.gov)] of 120 nsLTPs anchored in the soybean genome (**Supplementary Table 6**). Such GmnsLTPs belong to five distinct groups: GmnsLTP1, GmnsLTPg, GmnsLTPd, GmnsLTP2, GmnsLTPc and GmnsLTPe. The LTPf, LTPh, LTPj and LTPk groups were absent.

To evaluate the GmnsLTPs expression, two different approaches were used:

[I] *Baseline gene expression analysis*: it consisted of the detection (presence/absence) of transcripts of loci encoding GmnsLTPs. For this, RPKM-normalized $\log_2$-transformed counts for 14 soybean RNA-Seq libraries were processed including tissues/developmental stages: flower, root, young leaf, one centimeter pod, nodule, pod shell 10 DAF (days after flowering), pod shell 14 DAF, seed 10 DAF, seed 14 DAF, seed 21 DAF, seed 25 DAF, seed 28 DAF, seed 35 DAF, and seed 42 DAF. Such data came from the work by Severin et al. (2010), who provided an RNA Seq-Atlas with a record of high-resolution gene expression in a set of fourteen diverse tissues. These data are the informational base of the 'Expression' section of the Soybase (https://www.soybase.org/soyseq/) database, which is a reference in the integration of genetic and genomic data for advanced research in soybean.

[II] *Differential gene expression analysis*: this strategy accessed the transcriptional regulation of GmnsLTP loci in soybean under different (biotic and abiotic) stress conditions. The RNA-Seq libraries scrutinized here came from the Expression Atlas database, section '*Glycine max*' (https://www.ebi.ac.uk/gxa/experiments?species=glycine%20max). The mentioned platform employed DESeq2 v1.10.1 software for differential gene expression analysis. Genes with modulation of $-1 > Log_2FC > 1$ and $FDR < 0.05$ were

considered differentially expressed. Considering the GmnsLTPs, the assays addressed in the following articles were evaluated:

[a] Biotic stress – Transcriptome profiling of soybean (*G. max*) roots challenged with pathogenic and non-pathogenic isolates of *Fusarium oxysporum* (Lanubile et al. 2015) (Expression Atlas - E-GEOD-66861). The authors used RNA-seq analysis to investigate the molecular aspects of the interactions of a partially resistant soybean genotype with non-pathogenic/pathogenic isolates of *F. oxysporum* at 72 and 96 h post inoculation (hpi);

[b] Biotic stress – Genotypic variation of gene expression during the soybean innate immunity (Valdés-López et al. 2014) (Expression Atlas - E-GEOD-43463). In this work, a genome-wide transcriptome analysis of two soybean parental lines and two progeny lines treated for 30 min with the 1 mM flagellin flg22 peptide + 50 µg chitin was performed.

[c] Abiotic stress – Daytime soybean transcriptome fluctuations during water deficit stress reported by Rodrigues et al. (2015) in the soybean Expression Atlas (E-GEOD-69469). In this trial, the authors used control and drought-stressed soybean plants to verify the dynamic changes in gene expression during a 24-h time course.

[d] Abiotic stress – Comprehensive characterization and RNA-Seq profiling of the HD-Zip transcription factor family in soybean (*Glycine max*) during dehydration and salt stress by Belamkar et al. (2014; Expression Atlas - E-GEOD-57252). In this work, the authors identified members of the HD-Zip gene family in soybean cv. 'Williams 82', and characterized their expression under dehydration and salt stress.
