# Supplementary figures and images for: From Gene to Transcript and Peptide: A Deep Overview on Non-Specific Lipid Transfer Proteins (nsLTPs)
Source: Antibiotics (Basel). 2023 May 21;12(5):939. doi: 10.3390/antibiotics12050939 (PMC10215178; doi:10.3390/antibiotics12050939)

LTP1

LTP2

LTPg

LTPd

Unkown

LTPc

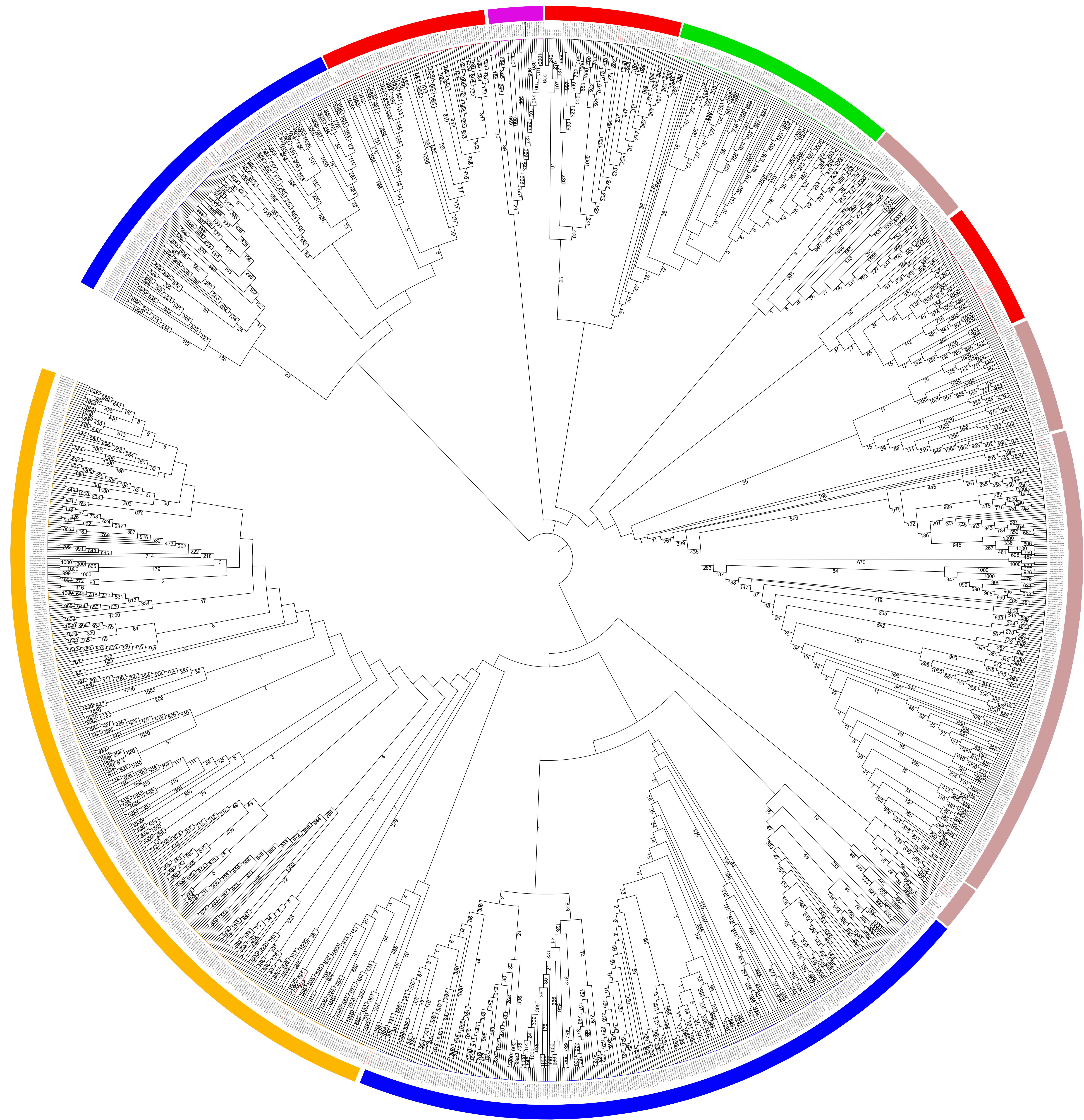

Supplement: Supplementary file 1 [file antibiotics-12-00939-s001.zip › Supplementary Figure 2.pdf]

**A**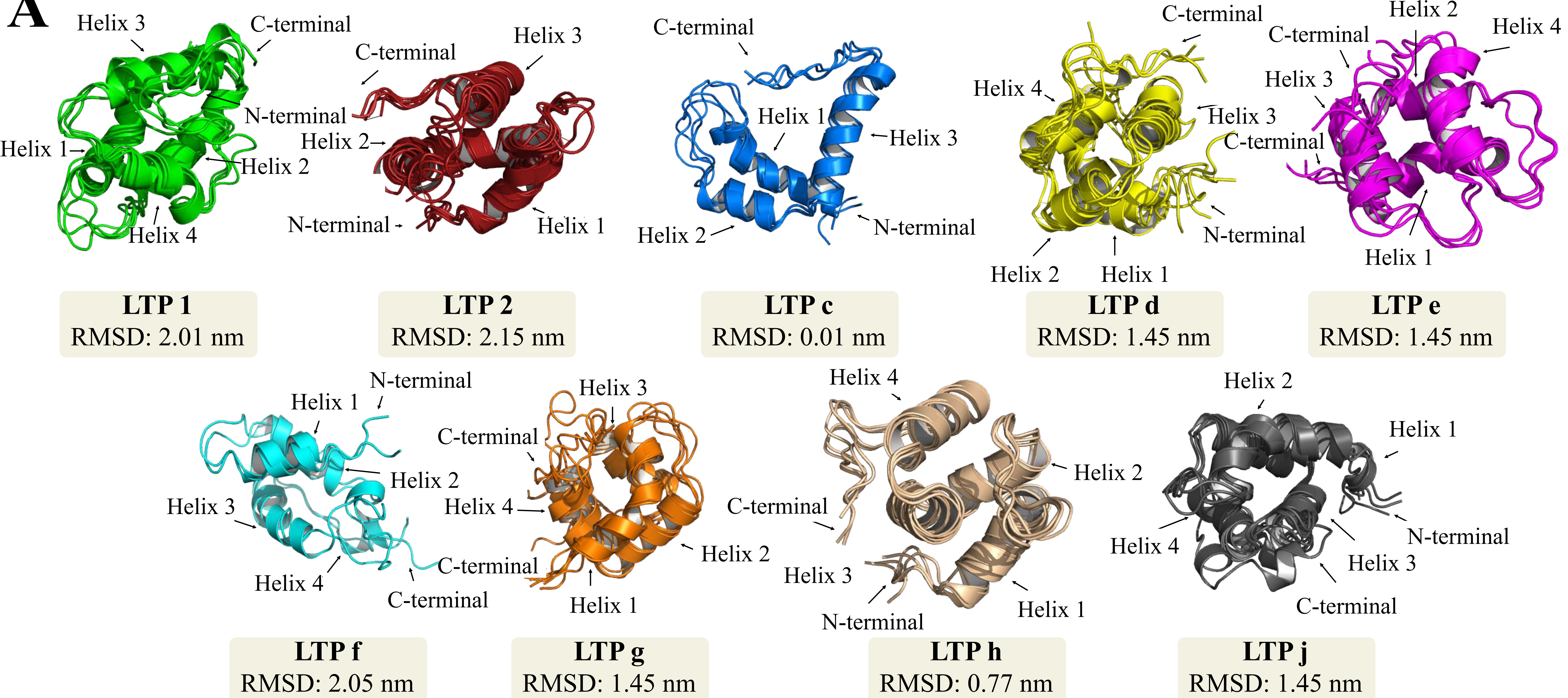**B**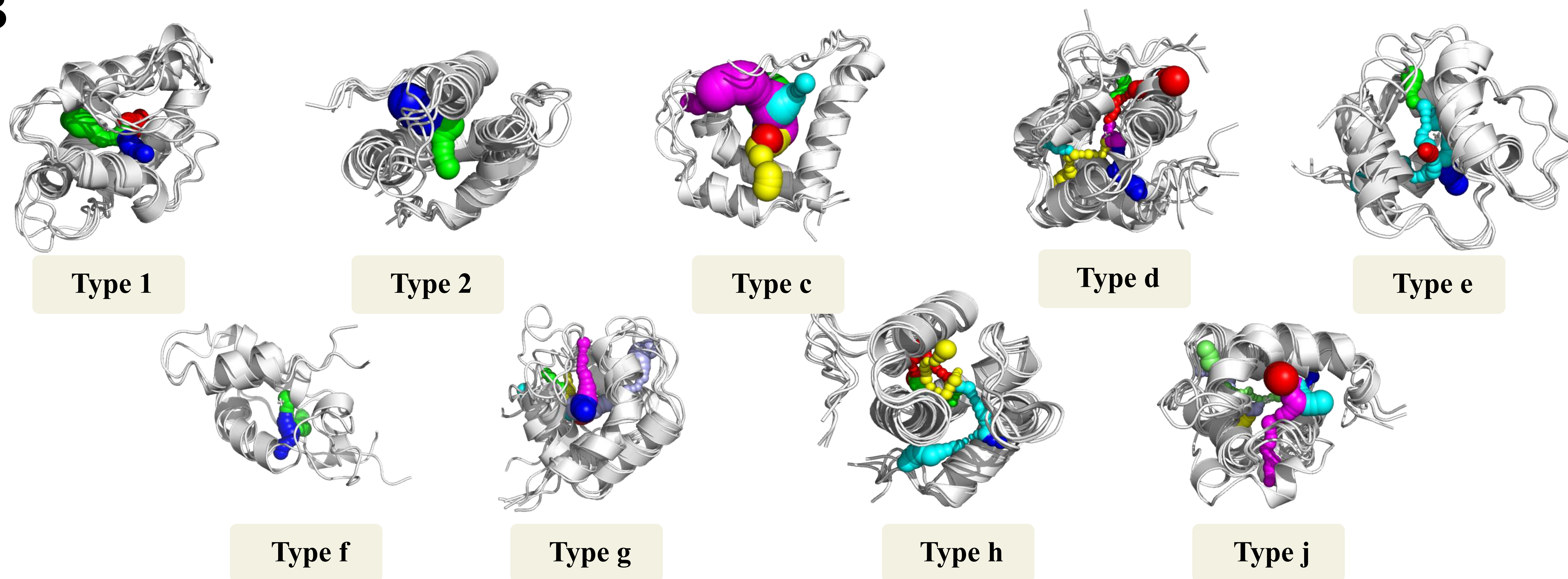

Supplement: Supplementary file 1 [file antibiotics-12-00939-s001.zip › Supplementary Figure 3A, B.pdf]

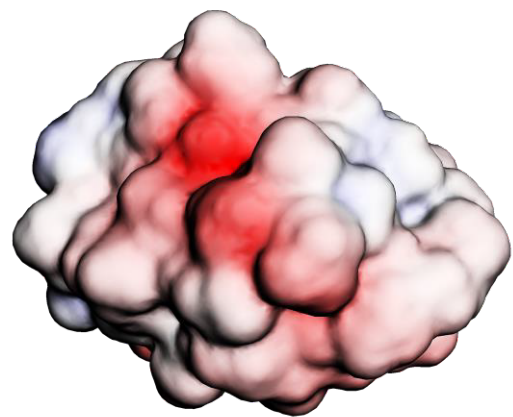

LTP1

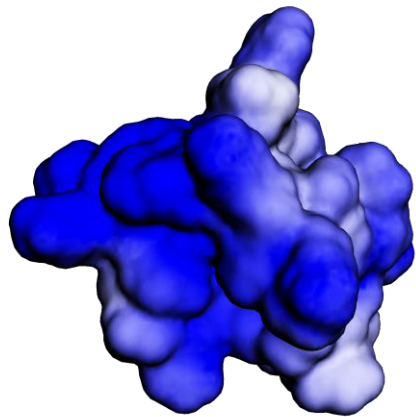

LTP2

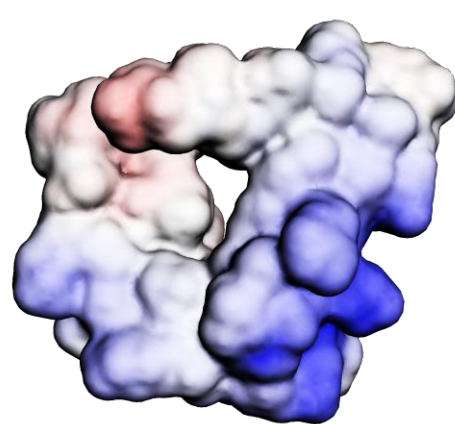

LTPc

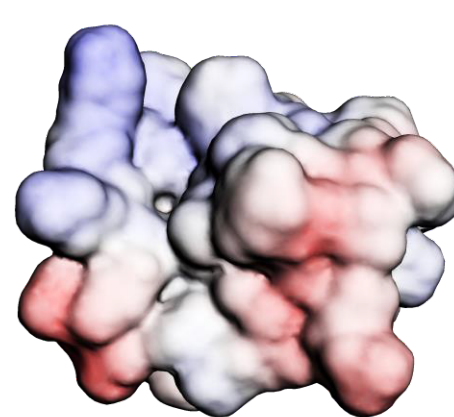

LTPd

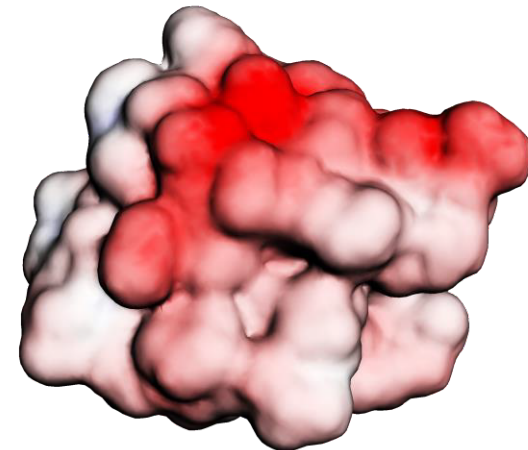

LTPe

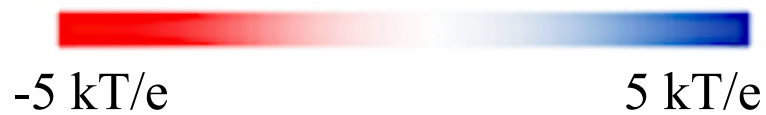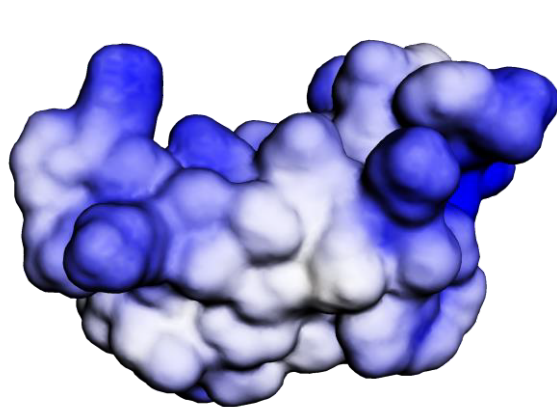

LTPf

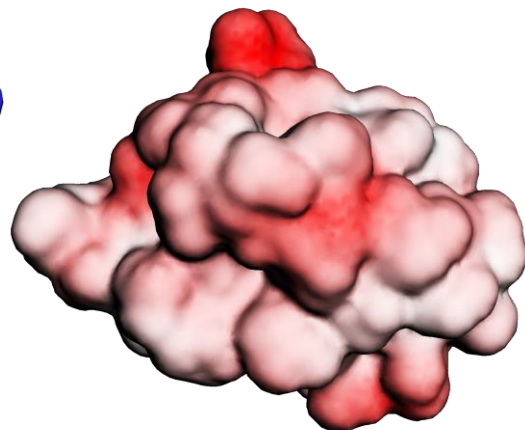

LTPg

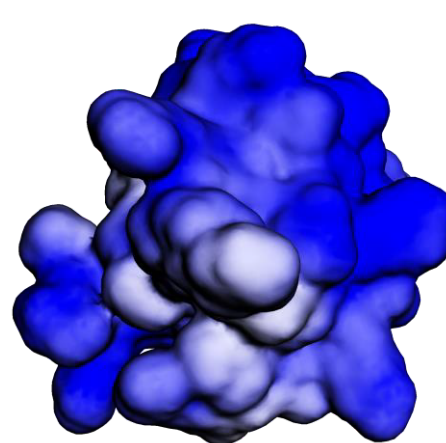

LTPh

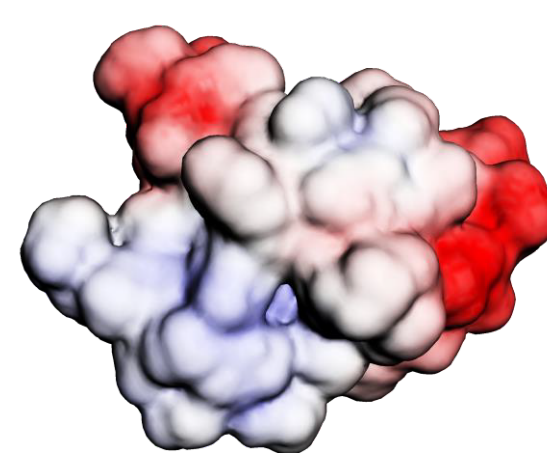

LTPj

Supplement: Supplementary file 1 [file antibiotics-12-00939-s001.zip › Supplementary Figure 4.pdf]
